# Aorto-Esophageal Fistula Secondary to Foreign Body Ingestion in Children: A Novel Treatment Approach and Comprehensive Narrative Review

**DOI:** 10.3390/children12121672

**Published:** 2025-12-09

**Authors:** Marco Di Mitri, Gabriele Egidy Assenza, Francesco Dimitri Petridis, Sara Schirru, Marta Agulli, Maria Elisabetta Mariucci, Emanuela Angeli, Edoardo Collautti, Tommaso Gargano, Mario Lima, Andrea Donti

**Affiliations:** 1 Pediatric Surgery Department, IRCCS Azienda Ospedaliero, Universitaria di Bologna, 40126 Bologna, Italy; 2 Pediatric Cardiology and Adult Congenital Heart Disease Program, Department of Cardio-Thoracic and Vascular Medicine, IRCCS Azienda Ospedaliero, Universitaria di Bologna, 40126 Bologna, Italy; 3 Pediatric Cardiac Surgery and Adult Congenital Heart Disease Program, Department of Cardio-Thoracic and Vascular Medicine, IRCCS Azienda Ospedaliero, Universitaria di Bologna, 40126 Bologna, Italy; 4 Cardiovascular Anesthesia, Department of Cardio-Thoracic and Vascular Medicine, IRCCS Azienda Ospedaliero, Universitaria di Bologna, 40126 Bologna, Italy

**Keywords:** aorto-esophageal fistula (AEF), foreign body (FB), button battery (BB), computed tomography (CT)

## Abstract

**Highlights:**

**What are the main findings?**

- Aorto-esophageal fistula (AEF) is a rare but highly lethal complication of foreign body ingestion in children, with button batteries responsible for 67% of cases and an overall 43% mortality rate.
- The presented case demonstrates the successful use of endovascular covered stent placement as an emergency, life-saving treatment option in a hemodynamically unstable child—an approach rarely reported in pediatrics.
- Review of 37 cases shows that symptoms are often nonspecific, but sentinel bleeding is the most crucial early warning sign.
- CT angiography is confirmed as the most accurate modality for detecting vascular complications, while X-rays identify the foreign body but often miss associated injuries.

**What are the implications of the main finding?**

- Early recognition of AEF—especially when minor bleeding follows button battery ingestion—is essential to prevent fatal outcomes.
- Endovascular stenting may serve as a rapid, less invasive alternative to emergent thoracotomy in selected pediatric cases, particularly in hemodynamically unstable children.
- A standardized diagnostic pathway including urgent CT angiography and multidisciplinary coordination could significantly improve survival.
- Public health measures, safer battery designs, and dedicated AEF registries are needed to reduce incidence, support early diagnosis, and guide evidence-based management.

**Abstract:**

***Background***: Aorto-esophageal fistula (AEF) is a rare but life-threatening condition in children following foreign body (FB) ingestion, with button batteries (BB) being the most dangerous. These batteries involve severe tissue necrosis due to chemical and electrical reactions, often leading to fistula formation and catastrophic hemorrhage. Appropriate treatment for AEF is still undefined. ***Method***: This report presents a novel case of AEF closure using a covered stent in a 4-year-old boy, complemented by a narrative review of 36 reported pediatric AEF cases from 1988 to 2024. ***Results***: The review revealed that BB ingestion accounted for 67% of AEF cases, with a high mortality rate of 43%, underscoring the critical nature of this condition. Early symptoms are often nonspecific, leading to delayed diagnoses, which worsen outcomes. Computed tomography (CT) is the key imaging modality for detecting vascular complications such as AEF, while X-ray may help identify the foreign body, but is often insufficient to assess associated injuries. While surgical repair remains the mainstay of treatment, minimally invasive techniques, such as endovascular approaches, are emerging as viable options. ***Conclusions***: This study highlights the need for heightened public awareness, safer battery designs, and prompt, multidisciplinary interventions to improve patient outcomes. Future research should focus on refining diagnostic protocols, evaluating innovative management strategies, and establishing comprehensive registries to inform evidence-based guidelines and optimize care.

## 1. Introduction

Aorto-esophageal fistula (AEF) is a rare but life-threatening complication resulting from foreign body (FB) ingestion in children [1]. Among the various causes of aorto-esophageal fistula, foreign body ingestion—especially button batteries—is the most frequent in the pediatric population. The most dangerous foreign bodies are button batteries (BB)—small, disc-shaped batteries commonly found in household items such as remote controls, toys, and hearing aids—which are easily swallowed by young children [2]. The pathophysiology of AEF in the context of button battery ingestion is multifaceted and complex [3]. When a BB is swallowed and dislodges in the esophagus, a cascade of events leads to tissue injury with the subsequent risk of esophageal perforation, bleeding, mediastinitis, and formation of fistulas with the trachea or aorta [4]. The electrical discharge of the battery creates a localized hydroxide-rich alkaline environment at its negative pole, which causes liquefactive necrosis of the esophageal tissue [5]. The process is further exacerbated by the release of heavy metals such as mercury, zinc, and silver from the battery, which are highly toxic to tissues [6]. Despite the increasing awareness of button battery injuries, the literature on pediatric aorto-esophageal fistula (AEF) remains limited to isolated case reports and small case series. No standardized diagnostic or therapeutic pathway exists, and the optimal management strategy is still debated. This represents a significant knowledge gap, especially considering the extremely high mortality associated with delayed diagnosis. To our knowledge, endovascular management of pediatric aorto-esophageal fistula has been rarely described, and we report one of the few cases treated successfully with covered stent placement following button battery ingestion. Because endovascular management in children has been reported only in exceptional cases, the contribution of each new experience is crucial to better understand its feasibility, risks, and potential role in the emergency treatment of pediatric AEF. The aim of this work is to present a complex pediatric case of AEF successfully treated with an endovascular covered stent and to integrate it within a narrative review of the literature. Our findings emphasize the importance of rapid multidisciplinary management and highlight endovascular stenting as a potential life-saving option in selected cases.

## 2. Case Report

A 4-year-old male (weight 14 kg) presented to the emergency department of a local hospital a month after urgent endoscopic removal of an accidentally ingested BB performed during a previous hospital admission.

The BB had been accidentally ingested 6 h before the endoscopic removal, and the child was discharged asymptomatic. The presenting symptoms at the second admission were bright red blood mixed with clots in his vomit, following a two-day history of coughing. Despite initially stable clinical conditions (HR 90 bpm, BP 102/60, SO_2_ 99%, RR 20 breaths/min, temperature 36 °C), an angio-CT scan was performed, confirming the diagnosis of aorto-esophageal fistula.

The patient was electively placed under general anesthesia and intubated before being helicopter-transferred to our center for further management. During the transport, the patient presented massive hematemesis and subsequent hemodynamic shock (severe tachycardia, hypotension).

A multidisciplinary team composed of cardiac anesthesiologists, pediatric surgeons, pediatric interventional cardiologists, and pediatric heart surgeons was rapidly dispatched before the patient’s arrival. The patient was directly transferred to the catheterization laboratory. Blood transfusion was administered along with colloid infusion. A focused, bedside echocardiogram showed normal biventricular function and no other structural or valvular anomalies. A Sengstaken–Blakemore (SB) Ch 16 tube was placed under radiologic guidance to control the esophageal bleeding. This maneuver was effective and likely life-saving. An “in-flight” multidisciplinary evaluation of the outside hospital CT exam showed a direct communication between the descending aorta immediately cranial to the diaphragmatic segment and the esophagus. Given the patient’s rapid hemodynamic deterioration, the anatomical location of the fistula, the urgent need for immediate hemorrhage control, and the high risk associated with emergent open thoracotomy in an unstable small child, endovascular exclusion was considered the safest and fastest option. The availability of an appropriately sized covered stent further supported this decision.

**Figure 1:**
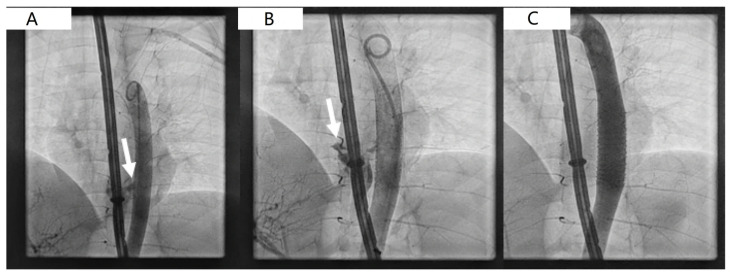
(**A**) Basal angiography showing spill over of contrast media from the aorta directly into the esophageal lumen through a relatively large fistulous pathway (white arrow). (**B**) Residual large gap after covered aortic stent implantation (white arrow) with significant opacification of esophageal lumen after aortic injection. (**C**) Complete sealing of aortic-esophageal fistula after high-pressure balloon stent dilation.

**Figure 2:**
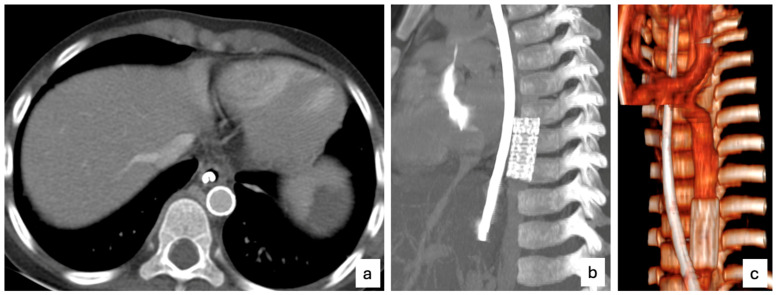
(**a**) Axial CT scan showing the covered stent correctly positioned in the distal thoracic aorta, with no evidence of extravasation, suggesting complete exclusion of the fistulous tract. The esophagus lies adjacent to the stented segment, without peri-esophageal collections. (**b**) Sagittal reconstruction demonstrating a well-expanded and properly apposed covered stent, with no signs of endoleak. (**c**) 3D volume-rendered reconstruction confirming the appropriate alignment and morphology of the aortic stent, with complete exclusion of the involved aortic segment and no parietal irregularities.

After patient sterile prepping, the left femoral artery was punctured, and a 4Fr hemostatic sheath was placed. Aortic angiography showed the presence of AEF located immediately above the diaphragm (Figure 1A and Online Supplementary Movie S1). A decision was made to pursue AEF closure using a covered stenting of the AEF feeding aortic segment. A Flexor 8Fr longsheath (Cook Medical, Bloomington, IN, USA) was advanced above the target landing zone using a 0.035 workhorse guidewire. A 38 mm covered stent, pre-mounted on a 10 mm balloon (Atrium, Getinge, Sweden, European Union) was implanted. An interim angiography showed persistent bleeding across the fistula with direct contrast media spill over in the esophagus and stomach (Figure 1B, Online Supplementary Movie S2), accordingly, a balloon remodeling of the stent using a high-pressure 12 × 40 mm balloon was accomplished with complete AEF exclusion (Figure 1C, Online Supplementary Movie S3). Special attention was paid to minimize the risk of spinal cord ischemia, which can occur due to coverage of intercostal arteries supplying the spinal cord. In our patient, the short length and distal location of the stent reduced this risk. The Atrium covered stent was chosen for its immediate availability and adequate radial force, despite its limited redilation capacity. Other devices were considered but deemed unsuitable due to the emergency setting, the small vessel caliber, and patient size. The femoral artery was surgically repaired as planned, given the small vessel diameter and to ensure hemostasis after sheath removal. After patient transfer to the intensive care unit, abdominal distension was noted with ultrasound evidence of a large gastric clot that was not retrievable using an endoscopic approach, leading to an exploratory laparotomy. During surgery, blood clots were found in the abdomen and removed, and a gastrotomy was performed to evacuate the large gastric clot and to reduce intra-abdominal pressure. The post-operative period was free of complications. The esophageal and gastric balloons of the Sengstaken–Blakemore tube were deflated respectively after 36 h and 48 h. On the fourth post-operative day, the SB tube was removed, and the patient was weaned off mechanical ventilation. In the post-operative period, a Replogle tube and a nasogastric tube were left on site for seven days. Postoperative management included broad-spectrum intravenous antibiotics, proton pump inhibitor therapy, and transfusion support as needed. The antiplatelet therapy was started due to the presence of the stent. The esophageal lesion was managed conservatively. Serial imaging and endoscopic follow-up demonstrated spontaneous healing without the need for surgical reinforcement. The patient was discharged home 42 days after the index procedure in good clinical condition after a follow-up CT scan confirming AEF exclusion, no stent fracture, and no aortic wall injury (Figure 2).

## 3. Methods

**Figure 3:**
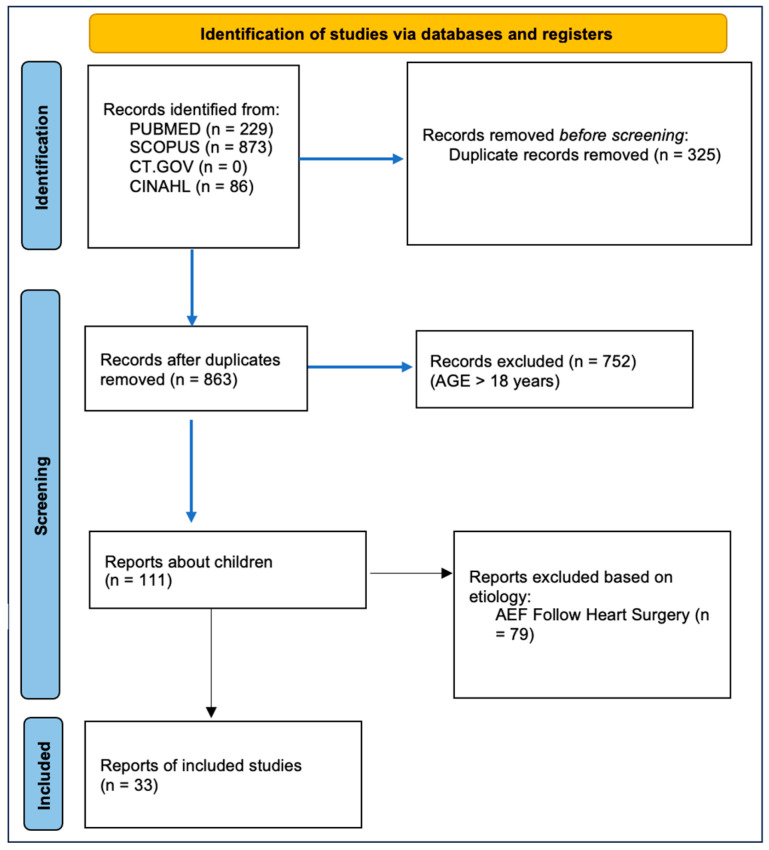
Preferred reporting items for systematic reviews and meta-analyses (PRISMA) flow chart of the analyzed studies.

Given the rarity of pediatric AEF and the heterogeneity of the available data, we conducted a narrative review supported by a systematically performed literature search. PubMed, Embase, Scopus, ClinicalTrials.gov, ICTRP, and CINAHL were queried in February 2024 using the terms “aorto-esophageal fistula”, “foreign body”, and “children”. (Figure 3-PRISMA steatment). Only pediatric cases caused by foreign body ingestion were included. No date restrictions were applied. Additional references were identified through cross-citation. Because available data consist almost exclusively of isolated case reports, a formal systematic review and meta-analysis were not feasible. All papers and abstracts retrieved from the initial search were independently evaluated for relevance by two reviewers. The relevant studies underwent a full-text review (Table 1). All stages of the review process, including screening, selection, and data extraction, were conducted independently by two reviewers. Any discrepancies were resolved through discussion and consensus. In addition to this, we present one case report from our experience, too.

**Table 1:** Studies involved in the review.

N	Authors	Year	Journal	FB Type	Age	Time to Removal	Time to AEF	Localization	Symptoms	Diagnosis	Treatment	Outcome
**1**	Grey et al. [7]	1988	Am J Forensic Med Pathol	Not sharp object	2 yo	-	-	Mid esophagus	Hematemesis, melena, shock	Autopsy	-	Deceased
**2**	Mcomas et al. [8]	1991	J of pediatric surgery	Sharp	8 mo	-	-	Mid esophagus	Hematemesis, melena	Surgery	Surgery	Alive
**3**	Jiraki et al. [9]	1996	Am J Forensic Med Pathol	Sharp	-	-	-	-	-	-	-	Deceased
**4**	Gilchrist et al. [10]	1997	Journal of pediatric surgery	Coin	2 yo	6 mo	6 mo	Proximal esophagus	Hematemesis, retrosternal pain	XR	Endoscopy (removal)	Deceased
**5**	Snajdauf et al. [11]	2005	Journal of pediatric surgery	Dieffenbachia	12 yo	6 h	38 d	Proximal esophagus	Hematemesis	Endoscopy	Endoscopy S.B tube Surgery	Alive
**6**	Stuth et al. [12]	2001	Anesthesiology	Coin	7 yo	-	-	-	Hematemesis, abdominal pain	Surgery	Surgery Foley	Alive
**7**	Hamilton et al. [13]	2008	Journal of pediatric surgery	2 BB	19 mo	24 h	9 d	Stomach, esophagus	Abdominal pain, dyspnea, anorexia, cough	XR	Endoscopy (removal) Surgery	Deceased
**8**	Mortensen et al. [14]	2010	Am J of emergency medicine	BB	14 mo	10 d	10 d	Mid esophagus	Fever, cough, vomit, black stools	Autopsy	-	Deceased
**9**	Hill et al. [15]	2010	Annals of vascular sugery	Not sharp object	9 yo	-	36 h	Mid esophagus	Chest pain and odynophagia	MRI	Endoscopy (removal)	Alive
**10**	Herrera et al. [16]	2010		BB	-	-	-	-	-	-	-	Deceased
**11**	Zhang et al. [17]	2011	A L.R and ontological surgery	Bone fish	17 yo	-	-	-	Hematemesis, retrosternal pain, fever	Unknow	Surgery	Deceased
**12**	Pae et al. [18]	2012	Anesthesiology	BB	4 yo	-	-	-	Hematemesis	XR	Surgery	Deceased
**13**	Spiers et al. [19]	2012	Journal of surgery	BB	10 mo	14 h	21 d	Distal esophagus	Dyspnea, hematemesis	XR	Endoscopy, Surgery	Alive
**14**	Ping Xi et al. [20]	2012	Surgical endoscopy	Chicken Bone	17 yo	7 d	7 d	Mid esophagus	Fever, Hematemesis, shock, dysphagia	XR	Surgery	Deceased
**15**	Pehlivan et al. [21]	2013	Journal of Forensic	Unknow	2 yo	7 d	7 d	Mid esophagus	Abdominal pain, Hematemesis	Autopsy	-	Deceased
**16**	Jayakumar et al. [22]	2015	Journal of pediatric surgery	BB	8 yo	-	-	-	Hematemesis	CT	Endoscopy Surgery S.B tube, Aortic stent	Alive
**17**	Barabino et al. [23]	2015	Digestive and liver disease	BB	22 mo	-	-	Mid esophagus	Hematemesis	Autopsy	-	Deceased
**18**	Taghavi et al. [24]	2015	Journal of Pediatrics and child health	BB	4 yo	14 d	-	Mid esophagus	Hematemesis, abdominal pain, melena	XR	Surgery	Deceased
**19**	Leinwand et al. [25]	2016	Gastrointestinal Endoscopy Clinics of North America	BB	16 mo	15 h	15 h	Stomach	Hematemesis, abdominal pain	XR, CT	Surgery	Deceased
				BB	2 yo	8 h	18 d	Distal esophagus	Chest pain, cough, vomit	Endoscopy	S.B tube	Deceased
				BB	18 mo	-	-	Mid esophagus	Hematemesis, pain	Autopsy	-	Deceased
**20**	Ventura et al. [26]	2016	The Am J of Forensic Medicine and Pathology	BB	18 mo	-	-	Mid esophagus	-	Autopsy	-	Deceased
**21**	Clarke et al. [27]	2016	Annals of thoracic surgery	Coin	6 yo	-	-	Proximal esophagus		XR, Angiography,	Surgery	Alive
**22**	Granata et al. [28]	2018	Journal of medical case reports	BB	3 yo	-	-	Stomach	Hematemesis	XR, CT	Surgery S.B tube Patch	Alive
**23**	Mahajan et al. [29]	2019	European journal of cardio-thoracic surgery	BB	3 yo	-	1 mo	Proximal Esophagus	Hematemesis	CT	Surgery	Alive
**24**	Bartkevies et al. [30]	2020	W J for pediatric and congenital heart surgery	BB	12 mo	-	17 d	Stomach	Hematemesis, melena	MRI, CT, Angiography	Surgery	Alive
**25**	Alrehelli et al. [31]	2021	Journal of case reports	BB	2 yo	16 h	9 d	Proximal Esophagus	Hematemesis	CT	Surgery	Alive
**26**	Muhieldin et al. [32]	2022	Journal of Cardiac Surgery	BB	17 mo	-	-	Esophagus	Lethargy, hematemesis, shock	XR	Surgery	Alive
**27**	Atlas et al. [33]	2021	Pediatric Surgery International	BB	-	-	14 d	Esophagus	Hematemesis	-	-	Alive
**28**	Wakimoto et al. [34]	2021	Saudi journal of Anesthesia	BB	17 mo	10 d	3 d	4	1	1		Alive
**29**	Lanzafame et al. [35]	2022	Diagnostics	BB	13 mo	-	-	Rectum	Hematemesis	CT	Surgery	Alive
**30**	Reddy et al. [36]	2023	Journal of Pediatric Surgery	Chiken bone	15 yo	4 d	4 d		Hematemesis	Endoscopy	S.B tube Surgery	Alive
				BB	3 yo	-	-	Esophagus	Hematemesis, melena	XR	Endoscopy (removal) S.B tube Surgery	Alive
				BB	2 yo	-	9 d	Esophagus	Hematemesis	Angio-CT	Surgery	Alive
**31**	Al-Taie et al. [37]	2023	Pediatrcs and international child health	BB	1 yo	-	-	Stomach	Hematemesis, Blood stool, vomit	Endoscopy	Surgery S.B tube	Alive
**32**	WhiteDzuro et al. [38]	2024	Journal of Pediatric Surgery Case Reports	BB	1 yo	-	-	Proximal Esophagus	Hematemesis	XR	Endoscopy (removal) Surgery Patch	Alive
				Coin	3 yo	-	-	Proximal Esophagus	Hematemesis	XR	Endoscopy Surgery	Deceased

BB, Button battery; CT, Computed tomography; FB, Foreign body; MRI, Magnetic resonance imaging; XR, X-ray.

## 4. Results

Thirty-two papers were analyzed, collecting 37 cases of AEF secondary to FB ingestion. The mean age at diagnosis was 5 years ± 4.4 (range 0.8–17).

In the analyzed reports, information about FB characteristics was found in 36 patients. Based on the FB characteristics, we identified the following groups: BB (n = 25; 67%), sharp objects (n = 5; 14%), and non-sharp objects (n = 7; 19%).

Information about the localization of the FB was found in 26 patients, reporting: proximal esophagus (n = 7; 27%), mid esophagus (n = 10; 38%), distal esophagus (n = 3; 12%), stomach (n = 5; 19%), rectum (n = 1; 4%).

Symptom data showed that AEF can manifest with a wide range of symptoms, including hematemesis, abdominal pain, melena, cough, shock, fever, chest pain, and dyspnea. We reported data about all 37 patients.

Information about the diagnostic workup was found in 35 patients, resulting in a predominant use of X-rays (n = 16; 45%), followed by CT scans (n = 7; 20%), endoscopy (n = 3; 7%), and MRI (n = 1; 3%). In 2 cases (5%), the diagnosis was reached during surgery, and in 7 cases (20%), AEF was diagnosed by autopsy. When stratifying cases by decade, CT usage increased markedly in recent years: 0/35 cases (1988–2014), 7/35 cases (2015–2024). This reflects historical limitations in CT availability, rather than underutilization.

Data about the treatment of 30 patients was collected. Among the reported cases, the fistulous communication most frequently involved the descending thoracic aorta, followed by the aortic arch and, rarely, the carotid artery.

Among 30 patients with available treatment data, Surgical repair was performed in 28/30 (94%), while 2/30 (6%) underwent patch reinforcement. Endovascular treatment was reported in 3 patients (10%), including 2 primary stent implantations and 1 hybrid repair.

Nine patients received temporary hemostatic measures (Sengstaken–Blakemore tube or aortic occlusion balloon). Of the 21 survivors, most (n = 19; 86%) underwent surgical repair, while a minority (N = 2; 9%) were successfully managed with endovascular techniques. Temporary hemostatic measures such as Sengstaken–Blakemore tube placement or aortic occlusion balloons (9 cases) were often used as bridges to definitive repair.

Data about the outcomes of all patients (n = 37) was collected, resulting in a high mortality rate (n = 16; 43%) (Table 2) [7,8,9,10,11,12,13,14,15,16,17,18,19,20,21,22,23,24,25,26,27,28,29,30,31,32,33,34,36,37,39].

## 5. Discussion

Aorto-esophageal fistula (AEF) is a rare but catastrophic complication following the ingestion of foreign bodies (FB), especially button batteries (BB), in children [39].

The increasing prevalence of BB has led to a rise in the incidence of accidental ingestions, making this a growing public health concern. BB ingestion occurs most frequently in children under six years old, with a peak incidence in toddlers who tend to explore their environment by placing objects in their mouths [2]. The small size and the shiny appearance of BB make them particularly attractive to children. In many cases, the ingestion is unwitnessed, leading to delays in the diagnosis and treatment. Several factors increase the risk of severe complications, including the size of the battery, the duration of esophageal impaction, and the location within the esophagus [35]. Larger batteries, typically bigger than 20 mm in diameter, are more likely to lodge in the esophagus and cause significant injury. Additionally, the longer the battery remains in the esophagus, the more likely the risk of severe complications, including AEF [3]. If a battery remains in the esophagus for several hours or days, the necrotic process can extend beyond the esophageal wall, leading to erosion of the adjacent aorta wall. The thinness of the posterior esophageal wall, combined with the proximity to the esophagus, makes this region particularly vulnerable to this type of injury [6]. Once the aortic wall is breached, a fistulous connection forms between the esophagus and the aorta, setting the stage for a catastrophic hemorrhage.

Based on the literature and our experience, suspected AEF should be managed with (i) immediate hemodynamic stabilization, eventually positioning the *Sengstaken–Blakemore tube,* and (ii) an urgent CT angiography. A proposed diagnostic pathway was added to improve clinical applicability. Clinical presentation of pediatric AEF typically includes a combination of hematemesis, melena, chest pain, or signs of hemodynamic instability. Among these, “sentinel bleeding” has emerged as the most critical early warning indicator, often preceding massive hemorrhage by hours or days. Rather than listing individual symptoms repetitively, the literature highlights the importance of recognizing this clinical pattern: a child with a history of button battery ingestion who develops even minimal upper gastrointestinal bleeding must be considered at high risk for AEF until proven otherwise. In our review, sentinel bleeding was reported in the majority of survivors, supporting its role as a prognostic factor and a trigger for urgent diagnostic imaging. This more integrated understanding of symptom evolution is essential for improving early diagnosis and reducing mortality. The diagnosis of BB ingestion is typically based on either witnessed ingestion or radiographic findings. A plain chest X-ray is typically the first diagnostic tool, revealing the characteristics of the FB: in case of BB ingestion, a double-rim or halo sign can distinguish BB from other radiopaque foreign bodies, such as coins [35]. In cases where AEF is suspected, further imaging investigations, including computed tomography (CT) angiography, may be necessary to assess the extent of the injury and to plan any surgical intervention [35,39]. The management of BB ingestion is still challenging, especially when complicated by AEF, requiring a multidisciplinary approach involving the pediatric surgeon, the endoscopist, the cardiothoracic surgeon, and the anesthesiologist. Once an AEF is suspected or diagnosed, prompt management is essential. aim to provide a comprehensive review of all cases of FB ingestion in children complicated with AEF. Although previous reports have described endovascular management of pediatric AEFs [40], our case adds to the literature by demonstrating successful use of a covered stent following button battery ingestion with documented radiologic healing and long-term survival. Compared with the other previously published pediatric cases managed with endovascular techniques, our approach shared similar indications—hemodynamic instability, distal thoracic location, and need for rapid bleeding control. However, differences were noted in device choice, vessel diameter, and timing of intervention. Although only a few pediatric AEF cases treated with endovascular stenting have been reported, and no standardized long-term surveillance protocol exists in the literature, regular imaging follow-up is advisable. Given the expected somatic growth of children and the potential mismatch between vessel diameter and stent size over time, periodic reassessment with CTA or MRA may be required to monitor stent position, aortic remodeling, and the possible need for future reintervention.

Our systematic review, which included 37 cases from the literature with high mortality (43%), highlights the severity and complexity of the management of this condition. Our review shows that most of the AEF cases resulted from BB ingestion (66%), underlying the extreme danger posed by these common household items. The high rate of AEF secondary to BB ingestion is due to the potential of the battery to rapidly cause tissue necrosis, evolving into aorta erosion, which can eventually result in fatal hemorrhage [4]. The mean age of the affected children was 5 years, which aligns with the well-documented tendency of younger children to explore their environment through their mouths. This highlights the importance of heightened vigilance and preventive strategies in households where young children live [41]. Given the relatively high prevalence of unwitnessed ingestions, awareness and clinical intuition are paramount to AEF early recognition because delayed diagnosis is associated with poor outcomes. The clinical presentation of AEF varies widely, with hematemesis being the most common symptom, reported in 86% of the cases. This is consistent with the typical presentation of AEF, where initial minor bleeding (“sentinel” bleeding) often precedes a massive hemorrhage [39]. However, the nonspecific nature of other symptoms, such as abdominal pain, melena, cough, and chest pain, makes early diagnosis challenging. Our findings show that symptoms can be misleadingly mild or absent, underscoring the need for a high index of suspicion, especially in cases of known or suspected FB ingestion.

The predominant use of X-rays for diagnosis in 50% of the cases highlights the limitations of the current diagnostic approaches. While X-rays are a valuable initial tool, they may not always reveal the full extent of the injury or confirm the presence of AEF. Computed tomography (CT) scans, which were used in 15% of the cases, offer superior detail and can better assess the extent of the injury. However, the relatively infrequent use suggests potential underutilization in acute settings, possibly due to resource constraints or inappropriate reluctance to expose young children to higher doses of X-ray. The high rate of AEF diagnosis at autopsy (20%) is an indirect indication for an aggressive and severe clinical course in many cases. Management of AEF is complex and typically requires a multidisciplinary approach. Rapid removal of the foreign body is of paramount importance [42]. The predominance of surgical procedures (93% of cases) underlines their critical role in managing this life-threatening condition. Thoracotomy and laparotomy remain the mainstays of treatment, allowing direct repair of the fistula and the associated vascular injuries. The use of the SB tube can be effective, and it has been reported in 25% of the cases. SB can induce immediate hemostasis in cases where surgical intervention is delayed, or during transport to a major center for surgical treatment [22].

Endovascular stenting offers immediate control of life-threatening hemorrhage and can serve as either definitive therapy or a bridge to surgical repair. However, limitations include potential endoleak, risk of spinal ischemia, and the challenge of long-term stent surveillance in growing children.

Despite these interventions, the mortality rate remains alarmingly high at 45%, and this is consistent with the severity of AEF and the possible rapid deterioration of the clinical conditions. The two cases managed with endovascular approaches, including endovascular patch placement, suggest that less invasive options may be viable in specific, carefully selected cases [28]. However, these approaches require further study to determine their efficacy and safety in broader clinical practice. After exclusion of the aortic lesion, attention must also be paid to the esophageal side of the fistula, where residual necrosis may lead to recurrent leakage or infection. Close endoscopic monitoring and staged repair should be considered if healing is incomplete.

Survival was associated with rapid hemostasis, multidisciplinary management, and early identification. Endovascular and hybrid techniques appear promising but remain limited to highly selected cases. There is a growing need for innovations in battery design that could reduce the risk of these severe injuries, such as coatings that prevent the generation of an electric current in a moist environment.

When comparing our case with previously published reports, several key considerations emerge. First, most pediatric AEFs described in the literature were managed through open surgical repair, which remains the standard approach in stable patients. However, mortality rates are particularly high in children presenting with massive hemorrhage or delayed diagnosis. Only a minority of cases report the use of endovascular techniques, generally as rescue therapy in unstable children. In our case, rapid hemodynamic deterioration and the anatomical characteristics of the fistula prompted a multidisciplinary decision to pursue emergency endovascular stenting, which allowed immediate bleeding control and avoided the high-risk scenario of emergent thoracotomy. This experience supports the potential role of endovascular exclusion as a life-saving option in selected pediatric patients, consistent with the limited but growing evidence available in the literature.

The findings suggest that expanding the use of CT angiography in suspected cases could improve diagnostic accuracy and reduce the incidence of fatal outcomes [39]. Future research should focus on identifying risk factors that may predict the development of AEF, optimizing diagnostic protocols to allow for earlier detection, and evaluating the efficacy of novel management strategies, including endovascular techniques and less invasive surgical options. Additionally, the creation of national and international registries for AEF cases could facilitate the collection of more comprehensive data, enabling more solid analysis and the development of evidence-based guidelines.

Future research should focus on the optimal timing of intervention and surveillance after battery removal. The concept of prophylactic endovascular reinforcement in high-risk necrotic lesions is intriguing but requires further validation. National and international registries could help standardize management and refine prognostic factors.

### Limitations and Further Research

This study has several limitations. First, as a single case report combined with a literature review, it cannot establish the superiority of one treatment over another. The available evidence is largely derived from case reports and small series, which limits the ability to perform quantitative comparisons or identify prognostic factors. Moreover, heterogeneity in the clinical presentation, timing of diagnosis, and management strategies across studies makes it difficult to draw definitive conclusions. Future research should focus on prospective multicenter data collection and long-term follow-up of pediatric patients who develop aorto-esophageal fistulas after foreign body ingestion. The establishment of international registries could help define standardized diagnostic and therapeutic algorithms and assess the long-term safety of endovascular devices in growing children.

## 6. Conclusions

The systematic review and case report underscore the critical challenges posed by aorto-esophageal fistulas (AEFs) following foreign body ingestion in children, particularly button batteries. The high mortality rate (43%) highlights the need for heightened awareness and improved management strategies. Looking ahead, future efforts should focus on prevention, such as public education campaigns targeting caregivers to emphasize the dangers of button battery ingestion and the importance of secure storage. Manufacturers must also innovate by designing safer batteries with child-resistant packaging and coatings to mitigate tissue injury.

On the clinical front, advancing diagnostic protocols, including the expanded use of CT angiography, may enable earlier detection of AEFs, thus improving outcomes. Additionally, exploring novel management strategies such as minimally invasive endovascular approaches holds promise, particularly for select cases where traditional surgery may be less feasible. Finally, establishing national and international registries dedicated to AEF cases will provide comprehensive data to inform evidence-based guidelines, optimize care pathways, and facilitate multidisciplinary collaboration. These steps are essential to reducing the incidence and improving outcomes for this life-threatening complication.

## Figures and Tables

**Table 2:** Children involved in an aorto-esophageal fistula after a foreign body (FB) ingestion: demographic data, type and localization of the FB, symptoms, diagnosis, treatment, and outcome.

***Patients***		
Sample, n (%)	N = 37	
Age, years	Mean: 5 (SD ± 4.4, range 0.8–17)	
***Trigger***		
Button battery, n (%)	25/36	67%
Sharp object, n (%)	5/36	14%
Non-sharp object, n (%)	***7***/36	29%
***Localization***		
Proximal esophagus, n (%)	7/26	27%
Mid esophagus, n (%)	10/26	38%
Distal esophagus, n (%)	3/26	12%
Stomach, n (%)	5/26	19%
Rectum, n (%)	1/26	4%
***Symptoms***		
Hematemesis, n (%)	32/37	86%
Abdominal pain, n (%)	9/37	25%
Melena, n (%)	6/37	17%
Cough, n (%)	5/37	14%
Shock, n (%)	4/37	11%
Chest pain, n (%)	4/37	11%
Fever, n (%)	1/37	3%
Dyspnea, n (%)	1/37	3%
***Principal diagnostic modality***		
XR, n (%)	17/35	45%
Autopsy, n (%)	7/35	20%
CT-scan, n (%)	7/35	20%
Endoscopy, n (%)	3/35	7%
Surgery, n (%)	2/35	5%
MRI, n (%)	1/35	3%
***Treatment***		
Surgery, n (%)	28/30	94%
Patch placement, n (%)	2/30	6%
***Sengstaken–Blakemore tube***		
Applied, n (%)	9/37	24%
Not applied, n (%)	28/37	76%
***Outcomes***		
Alive, n (%)	21/37	57%

CT, Computed tomography; MRI, Magnetic resonance imaging; SD, Standard deviation.

## Data Availability

No new data were created or analyzed in this study.

## Supplementary Materials

The following supporting information can be downloaded at: https://www.mdpi.com/article/10.3390/children12121672/s1, Movie S1. Pigtail-directed aortic injection showing large opacification of esophageal lumen through a direct fistulous pathway between descending aorta and the esophagus. Movie S2. Residual and significant fistula after covered aortic stent implantation with contrast media spill over to the esophagus and stomach. Movie S3. Complete exclusion of aortic-esophageal fistula after high-pressure balloon dilation of the aortic stent.
